# Susceptibility to tulathromycin in *Mannheimia haemolytica* isolated from feedlot cattle over a 3-year period

**DOI:** 10.3389/fmicb.2013.00297

**Published:** 2013-10-09

**Authors:** Trevor W. Alexander, Shaun Cook, Cassidy L. Klima, Ed Topp, Tim A. McAllister

**Affiliations:** ^1^Lethbridge Research Centre, Agriculture and Agri-Food CanadaLethbridge, AB, Canada; ^2^Southern Crop Protection and Food Research Centre, Agriculture and Agri-Food CanadaLondon, ON, Canada

**Keywords:** *Mannheimia haemolytica*, tulathromycin, antimicrobial resistance, feedlot, cattle

## Abstract

*Mannheimia haemolytica* isolated from feedlot cattle were tested for tulathromycin resistance. Cattle were sampled over a 3-year period, starting 12 months after approval of tulathromycin for prevention and treatment of bovine respiratory disease. Nasopharyngeal samples from approximately 5,814 cattle were collected when cattle entered feedlots (*N* = 4) and again from the same cattle after ≥60 days on feed. The antimicrobial use history for each animal was recorded. *Mannheimia haemolytica* was isolated from 796 (13.7%) entry samples and 1,038 (20.6%) ≥ 60 days samples. Of the cattle positive for *M. haemolytica*, 18.5, 2.9, and 2.4% were administered therapeutic concentrations of tulathromycin, tilmicosin, or tylosin tartrate, respectively. In addition, 13.2% were administered subtherapeutic concentrations of tylosin phosphate in feed. In years one and two, no tulathromycin-resistant *M. haemolytica* were detected, whereas five isolates (0.4%) were resistant in year three. These resistant isolates were collected from three cattle originating from a single pen, were all serotype 1, and were genetically related (≥89% similarity) according to pulsed-field gel electrophoreses patterns. The five tulathromycin-resistant isolates were multi-drug resistant also exhibiting resistance to oxytetracycline, tilmicosin, ampicillin, or penicillin. The macrolide resistance genes *erm*(42), *erm*(A), *erm*(B), *erm*(F), *erm*(X) and *msr*(E)-*mph*(E), were not detected in the tulathromycin-resistant *M. haemolytica*. This study showed that tulathromycin resistance in *M. haemolytica* from a general population of feedlot cattle in western Canada was low and did not change over a 3-year period after tulathromycin was approved for use in cattle.

## INTRODUCTION

Bovine respiratory disease (BRD) continues to be a health issue for feedlots in North America. The disease results in direct economic losses due to morbidity and mortalities and indirect losses resulting from reduced feed efficiency and meat quality (Duff and Galyean, 2007). Cattle exhibiting signs of BRD can be infected by more than one causative agent, but *Mannheimia haemolytica* is consistently detected as the primary bacterial agent of BRD (Zecchinon et al., 2005). In feedlots, BRD is primarily managed through administration of vaccines and antimicrobial agents. Vaccination is typically for viruses and to a lesser extent, for bacteria such *as M. haemolytica*, although efficacy has been variable (Larson and Step, 2012). In contrast, antimicrobial agents such as ceftiofur, florfenicol, tulathromycin, and tilmicosin have been shown to reduce morbidity when used metaphylactically on high-risk cattle entering feedlots (Taylor et al., 2010).

Macrolides are used extensively in feedlot production systems at subtherapeutic concentrations to promote growth and therapeutic concentrations to treat or prevent bacterial infections, including those that are BRD-associated (Marshall and Levy, 2011). Macrolides consist of a core macrolactone ring that binds to the large subunit of the ribosome and blocks bacterial protein synthesis (Poehlsgaard and Douthwaite, 2005). Differences in the ring structure can affect pharmacokinetic/pharmacodynamic properties and have been the basis for the development of new macrolide derivatives over the years (Godinho, 2008). Tulathromycin is a macrolide that has three amine rings and is further sub-classified as a triamilide. In 2006, tulathromycin was approved for use in Canada to treat and prevent BRD in high-risk cattle entering feedlots (Schunicht et al., 2007). Compared to other antimicrobial agents, tulathromycin has been shown to be equivalent or more efficacious in therapeutic treatment of feedlot cattle displaying BRD (Nutsch et al., 2005; Skogerboe et al., 2005) and in preventing cases of BRD after metaphylactic administration to high-risk cattle (Booker et al., 2007; Van Donkersgoed et al., 2008). Consequently it has become widely used by the feedlot industry.

Recent studies have characterized genes in* M. haemolytica* that provide cross-resistance to tulathromycin and other macrolides (Desmolaize et al., 2011; Michael et al., 2012a; Rose et al., 2012). Resistance to antibiotics commonly used for BRD poses animal health and economic concerns. Despite this, few epidemiological studies have been conducted in North America to monitor macrolide resistance in veterinary pathogens. We have previously shown macrolide resistance to be low in *M. haemolytica* isolated from randomly selected feedlot cattle, although in this study isolates were collected over a short period of time (Klima et al., 2011). In a multi-year study, it was reported that susceptibility to tulathromycin and tilmicosin decreased over a 5-year period in *M. haemolytica* isolated from diseased or deceased cattle in Canada and the United States (Portis et al., 2012). However, animal history, including antimicrobial use, was not reported and isolates from healthy cattle were not included in the analysis (Portis et al., 2012). The purpose of the current study was to evaluate tulathromycin resistance in *M. haemolytica* isolated from cattle with a known history of antimicrobial use over a 3-year period.

## MATERIALS AND METHODS

### ANIMALS

As part of a longitudinal surveillance study monitoring resistance in bovine fecal and respiratory bacteria (Klima et al., 2011; Alexander et al., 2013), *M. haemolytica* were isolated from feedlot cattle with a known history of antimicrobial use. Nasopharyngeal samples were collected from cattle at four commercial feedlots in southern Alberta, Canada between September, 2007 and May, 2010. A subset of cattle (a random selection of approximately 10% of animals from 30% of feedlot pens) housed within the feedlots were enrolled in the study. The cattle were sampled upon entry into feedlots and again after ≥60 days on feed. In total, 5,814 cattle were enrolled in the study. Antimicrobial administration was recorded for all cattle, as described previously (Klima et al., 2011). Sampling took place from September, 2007 to August, 2008 (year 1), September, 2008 to August, 2009 (year 2), and September, 2009 to May, 2010 (year 3). These dates correspond to 2, 3, and 4 years after tulathromycin was approved for use in Canada (Health Canada, 2006).

### BACTERIAL ISOLATION

Double guarded nasal swabs were used to sample the nasopharynx of cattle. Swabs were stored in Cary Blair transport medium (BD Canada, Inc., Mississauga, ON, Canada) at 4°C prior to processing, as described by Klima et al. (2011). Briefly, swabs were vortexed in 0.7 mL of brain heart infusion (BHI) broth, and a 100-μL aliquot of the suspension was cultured on tryptic soy agar (TSA) plates containing 5% sheep blood and 15 μg/mL of bacitracin (Dalynn Biologicals, Inc., Calgary, AB, Canada) at 37°C for 24 h (Catry et al., 2006). Isolates displaying morphology indicative of *Mannheimia* were subcultured (conditions as above) from each plate and tested for catalase and oxidase. Isolates with the typical colony morphology and that were both catalase and oxidase positive were tentatively identified as *M. haemolytica. *Identity was confirmed using a multiplex PCR assay (Alexander et al., 2008). Up to three confirmed isolates from each positive animal were stored at -80°C in BHI broth containing 20% glycerol.

### ANTIMICROBIAL SUSCEPTIBILITY

Isolates of *M. haemolytica* (*N* = 4,548) were screened for tulathromycin susceptibility by plating onto BHI plates supplemented with 2 μg/mL tulathromycin, followed by incubation at 37°C for 24 h. A concentration of 2°μg/mL was selected based on a previous study reporting 97% of bovine *M. haemolytica* field isolates having tulathromycin minimum inhibitory concentrations (MICs) of ≤2 μg/mL (Godinho, 2008). Tulathromycin was kindly provided by Pfizer Animal Health (now Zoetis) and prepared according to the manufacturer’s instructions. Isolates that grew on tulathromycin-supplemented BHI plates were further tested for antimicrobial susceptibility using a commercially available broth microdilution panel (Bovine/Porcine with Tulathromycin MIC Format, Sensititre; Trek Diagnostic Systems, Cleveland, OH, USA). A list of the antimicrobial agents utilized and the range of concentrations tested are presented in **Table 1**. In addition to these, sulphadimethoxine and trimethoprim/sulfamethoxazole were included in the panel at single breakpoint concentrations of 256 and 2/38 μg/mL, respectively. Bacterial growth was assessed by visual assessment and MICs were defined according to recommendations provided in the Clinical and Laboratory Standards Institute document M31-A3 (CLSI, 2008). Clinical and Laboratory Standards breakpoints were not available for clindamycin, neomycin, penicillin, tiamulin, sulphadimethoxine, trimethoprim/sulfamethoxazole or tylosin tartrate. Therefore, susceptibility designations for these drugs were not assigned. Exceptions to this are cases where isolates exhibited a high MIC for neomycin (MIC 32 μg/ml) or penicillin (MIC 8 μg/ml) and harbored the corresponding resistance determinant, *aphA-*1 or* bla*_ROB-1_, respectively.

**Table 1:** The MICs of *M. haemolytica* isolated from the nasopharynx of feedlot cattle^a^

Antibiotic (concentrations tested, μg/mL)	Isolate				
	32A	32B	32C	50A	55A
Ampicillin (0.25, 0.5, 1, 2, 4, 8, 16^*^)	0.25	0.25	0.25	0.25	>16
Ceftiofur (0.25, 0.5, 1, 2, 4, 8^b^)	0.25	0.25	0.25	0.25	0.5
Chlortetracycline (0.5, 1, 2, 4, 8)	4	4	2	2	2
Clindamycin (0.25, 0.5, 1, 2, 4, 8, 16)	16	16	16	16	16
Danofloxacin (0.12, 0.25^b^, 0.5, 1)	0.12	0.12	0.12	0.12	0.12
Enrofloxacin (0.12, 0.25, 0.5, 1, 2^b^)	0.12	0.12	0.12	0.12	0.12
Florfenicol (0.25, 0.5, 1, 2, 4, 8^b^)	1	1	1	1	0.5
Gentamycin (1, 2, 4, 8, 16)	2	2	2	2	2
Neomycin (4, 8, 16, 32^*^)	>32	>32	>32	>32	>32
Oxytetracycline (0.5, 1, 2, 4, 8^b^)	>8	>8	>8	>8	>8
Penicillin (0.12, 0.25, 0.5, 1, 2, 4, 8^*^)	0.12	0.12	0.25	0.25	>8
Spectinomycin (8, 16, 32, 64)	32	32	32	32	32
Tiamulin (0.5, 1, 2, 4, 8, 16, 32)	32	32	32	32	16
Tilmicosin (4, 8, 16, 32^b^, 64)	64	64	64	>64	>64
Tulathromycin (1, 2, 4, 8, 16, 32, 64^b^)	64	64	64	64	64
Tylosin tartrate 0.5, 1, 2, 4, 8, 16, 32)	>32	>32	>32	>32	>32

a Isolates were first screened for reduced tulathromycin susceptibility by culturing onto BHI plates supplemented with tulathromycin (2 μg/mL).

b Breakpoints defining resistance (CLSI, 2008).

* Resistance breakpoint defined by authors.

### PCR DETECTION OF ANTIMICROBIAL RESISTANCE GENES

Isolates of *M. haemolytica* that grew on BHI plates supplemented with 2 μg/mL of tulathromycin were analyzed for resistance genes using PCR. A description of genes, primers, and annealing temperatures are found in **Table 2**. Colonies of *M. haemolytica* were heat lysed (98°C for 4 min) and centrifuged (16,000 × *g*; 4 min), and the supernatant (1 μL) was added to the PCR mixture as DNA template. PCR were performed using 1× Qiagen HotStarTaq Plus Master Mix (Qiagen Inc.) on a MasterCycler thermal cycler (Eppendorf). Twenty microliters of product was visualized on a 1.5% (wt/vol) agarose gel following electrophoresis and staining with ethidium bromide. All PCR were run with positive controls (plasmids with sequence-verified genes) for respective gene determinants and negative controls containing water in place of a DNA template.

**Table 2:** Primers used to screen for antimicrobial resistance genes in *M. haemolytica* isolated from feedlot cattle.

Resistance phenotype^a^	Resistance gene	Primer sequences 5′–3′	Annealing (°C)	Reference
Amp, Pen	*bla*_ROB-1_	AATAACCCTTGCCCCAATTC	60	Klima et al. (2011)
		TCGCTTATCAGGTGTGCTTG		
Neo	*aphA-1*	TTATGCCTCTTCCGACCATC	54	Klima et al. (2013)
		GAGAAAACTCACCGAGGCAG		
Tet	*tet(*H)	ATACTGCTGATCACCGT	60	Klima et al. (2011)
		TCCCAATAAGCGACGCT		
Til, Tul	*erm*(A)	GAAATYGGRTCAGGAAAAGG	55	Chen et al. (2007)
		AAYAGYAAACCYAAAGCTC		
	*erm*(B)	GATACCGTTTACGAAATTGG	58	Chen et al. (2007)
		GAATCGAGACTTGAGTGTGC		
	*erm*(F)	CGACACAGCTTTGGTTGAAC	56	Chen et al. (2007)
		GGACCTACCTCATAGACAAG		
	*erm(*X)	GAGATCGGRCCAGGAAGC	58	Chen et al. (2007)
		GTGTGCACCATCGCCTGA		
	*erm*(42)	GGGTGAAAAGGGCGTTTATT	60	Kadlec et al. (2011)
		ACGTTGCACTTGGTTTGACA		
	*msr*(E)-*mph*(E)	TACCGGAACAACGTGATTGA	60	Kadlec et al. (2011)
		GAAGGGTTACGCCAGTACCA		

a Amp, ampicillin; Neo, neomycin; Pen, penicillin; Tet, tetracycline; Til, tilmicosin; Tul, tulathromycin.

### SEROTYPING AND PULSED-FIELD GEL ELECTROPHORESIS

*Mannheimia haemolytica* that grew on BHI plates supplemented with 2 μg/mL of tulathromycin were further characterized by serotyping and PFGE. Serotyping was performed using the rapid plate agglutination procedure as described by Frank and Wessman (1978). For PFGE, macro-restriction digest was performed according to Klima et al. (2010), using *Sal*I (New England Biolabs) restriction enzyme.

## RESULTS

### ISOLATION OF *MANNHEIMIA HAEMOLYTICA* AND ADMINISTRATION OF MACROLIDE ANTIMICROBIAL AGENTS

A total of 5,814 cattle were enrolled in the study and sampled upon entry into feedlots (**Table 3**). Of these, 5,036 were sampled a second time after ≥60 days on feed. *M. haemolytica* was isolated from 796 (13.7%) to 1,038 (20.6%) of entry and ≥60 days samples, respectively. In total, 1,688 cattle were positive for *M. haemolytica* at entry, ≥60 days on feed, or at both samplings. Of the cattle positive for *M. haemolytica* upon entry, only 146 were positive at the second sampling. There were 403, 821, and 464 cattle positive for *M. haemolytica* during years 1, 2, and 3 of the study, respectively (**Table 4**). From these cattle, 862, 2,342, and 1,344 *M. haemolytica* isolates were isolated in years 1–3 (**Table 4**).

**Table 3:** History of macrolide use in feedlot cattle that were positive for *M. haemolytica* over a 3-year period.

	Number of cattle^a^	
	Entry	≥60 dayson feed	Total
Cattle sampled	5,814	5,036	10,850
Positive for *M. haemolytica*	796	1,038	1,834
Administered tulathromycin^b^	259	53	312
Administered tilmicosin^c^	43	6	49
Administered tylosin tartrate^d^	41	0	41
Administered tylosin phosphate^e^	0	223	223

a The majority of cattle were sampled at both entry into feedlots and after ≥60 days on feed. Entry administration of antimicrobial agents denotes metaphylactic treatment within 2 days of arrival to feedlots. The ≥60 days on feed administration of antimicrobial agents denotes therapeutic or subtherapeutic treatment while placed in feedlots. Antimicrobial administration is shown only for cattle positive for *M. haemolytica* and is represented across entry and ≥60 days on feed samples.

b Subcutaneous (2.5 mg/kg BW). Used for prevention and treatment of BRD.

c Subcutaneous (10 mg/kg BW). Used for prevention and treatment of BRD.

d Subcutaneous (29 mg). Used for preventing abscesses at implant site.

e In feed (11 mg/kg dry matter). Used to prevent liver abscesses.

**Table 4:** Number of cattle and *M. haemolytica* analyzed by year of the study^a^.

	Year 1	Year 2	Year 3
Cattle positive for *M. haemolytica*	403	821	464
Cattle positive for Tul^R^ *M. haemolytica*	0	0	3 (0.6%)
*M. haemolytica* screened	862	2342	1344
Tul^R^ *M. haemolytica*	0	0	5 (0.4%)

a Number of cattle is represented across entry and ≥60 days samples. One to three *M. haemolytica* isolates per animal were tested.

*Tul^R^, tulathromycin-resistant.*

Combined, 43% of cattle positive for *M. haemolytica* at entry were metaphylactically administered doses of the macrolides tulathromycin (*N* = 259), tilmicosin (*N* = 43), or tylosin tartrate (*N* = 41) within 2 days of arrival (**Table 3**). Fifty nine cattle received therapeutic administration of a macrolide during placement, with the majority receiving tulathromycin (*N* = 53). The in-feed macrolide tylosin phosphate was administered subtherapeutically to 223 (13.2%) of the cattle carrying *M. haemolytica*. Within the cattle administered tulathromycin, 9 were also treated with tilmicosin and 45 were administered tylosin phosphate in feed (data not shown).

### *MANNHEIMIA HAEMOLYTICA* WITH REDUCED TULATHROMYCIN SUSCEPTIBILITY

Of the 4,548 *M. haemolytica* isolates tested for reduced susceptibility to tulathromycin, only five grew on BHI plates supplemented with 2 μg/mL of tulathromycin (**Table 4**). These isolates were cultured from three cattle housed in the same pen during the third year of the study (**Table 5**). Isolates 32A, 32B, and 32C were cultured from animal A028 and were the only *M. haemolytica* isolated and stored from this individual. Isolate 50A was cultured from animal A156. A second isolated from this individual did not exhibit reduced susceptibility to tulathromycin. Isolate 55A was cultured from animal A877 and was the only strain recovered from this animal. In all instances, isolates with reduced susceptibility were obtained from samples collected at ≥60days on feed, on February 10, 2010. A complete history of antimicrobial use for these three individuals is presented in **Table 5** with all three having been administered metaphylactic doses of tulathromycin

**Table 5:** History of cattle colonized with tulathromycin-resistant *M. haemolytica*^a^.

Animal number	Days enrolled in study	*M. haemolytica* isolated ≥60 days on feed	Antimicrobial use			
			Antimicrobial (dose administered)	Administration route	Day of administration	Day of withdrawal
A028	84	32A, 32B, 32C	Tulathromycin (2.5 mg/kg BW)^b^	Subcutaneous	1	-
			Chlortetracycline (35 mg/kg diet DM)^c^	In feed	1	84
			Chlortetracycline (6 g/head/day)^d^	In feed	1	22
			Monensin sodium (25 mg/kg diet DM)^e^	In feed	1	84
A156	82	50A	Tulathromycin (2.5 mg/kg BW)	Subcutaneous	1	-
			Chlortetracycline (35 mg/kg diet DM)	In feed	1	82
			Monensin sodium (25 mg/kg diet DM)	In feed	1	82
A877	86	55A	Tulathromycin (2.5 mg/kg BW)	Subcutaneous	1	-
			Chlortetracycline (35 mg/kg diet DM)	In feed	1	86
			Monensin sodium (25 mg/kg diet DM)	In feed	1	86

a All cattle were housed in the same pen and sampled on February 10, 2010.

b Used for prevention of BRD.

c Used to prevent liver abscesses.

d Used to prevent *Histophilus somni* infection.

e Used to promote growth and control coccidiosis.

### CHARACTERIZATION OF *MANNHEIMIA HAEMOLYTICA* ISOLATES WITH REDUCED TULATHROMYCIN SUSCEPTIBILITY

The five *M. haemolytic* isolates capable of growth on BHI plates supplemented with 2 μg/mL of tulathromycin were further characterized for susceptibility against a panel of antimicrobial agents (**Table 1**). Each of the isolates grew in 256 μg/mL sulphadimethoxine, but failed to grow in 2/38 μg/mL trimethoprim/sulfamethoxazole. With the exception of isolate 55A, MICs were similar among isolates. All isolates were resistant to neomycin, oxytetracycline, tilmicosin, and tulathromycin. In addition to these, isolate 55A was also resistant to ampicillin and penicillin.

**FIGURE 1:**
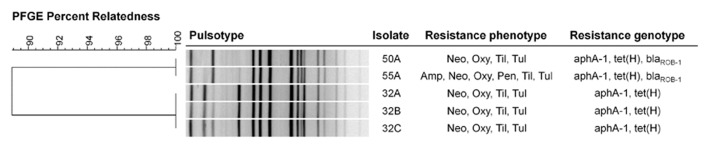
**Genetic relatedness and resistance profiles of tulathromycin-resistant *Mannheimia haemolytica* collected from feedlot cattle.** The dendrogram was created using UPGMA clustering of Dice coefficient values. Similarity matrix was based on band-matching analysis, optimization and tolerance settings of 1.0 and 1.5%, respectively. Resistance genotype was based on detection of resistance genes by PCR. Neo, neomycin; Oxy, oxytetracycline; Til, tilmicosin; Tul, tulathromycin, Amp, ampicillin.

Each of the isolates harbored *aphA-1* and *tet*(H), conferring resistance to neomycin and oxytetracycline, respectively (**Figure 1**). Isolates 50A and 55A also harbored *bla*_ROB-1_, which encodes resistance to ampicillin and penicillin, even though only 55A exhibited phenotypic resistance to these antibiotics. None of the macrolide resistance genes that were screened for were detected by PCR in any of the isolates.

All five *M. haemolytica* isolates were serotype 1. Upon PFGE analysis, a dendrogram of the isolates revealed two clusters (**Figure 1**). Isolates 32A, 32B, and 32C originating from animal A028 exhibited identical pulsotypes forming one cluster whereas isolates 50A and 55A from animals A156 and A877 had similar pulsotypes and formed a second cluster.

## DISCUSSION

Across the four feedlots, the prevalence of *M. haemolytica* carriage at entry (13.7%) was similar to previous studies that sampled healthy feedlot cattle (Klima et al., 2011; Fulton et al., 2002). Only 146 cattle were positive for *M. haemolytica* at both samplings and a large number of cattle initially positive at entry became negative at ≥60days of feed. Despite this, overall prevalence increased at the ≥60 days on feed time point (20.6%). Active colonization of the nasal mucosa by *M. haemolytica* has been shown to fluctuate in feedlot cattle (Magwood et al., 1969) and this may explain why the majority of cattle shedding at entry or ≥60 days on feed were only positive at one of the sampling times, and not the other. The tonsillar crypts have been suggested to be a reservoir for *M. haemolytica* (Frank and Briggs, 1992) and isolation from the tonsils is possible when nasal samples are negative (Frank et al., 1994). It has been suggested that sampling at the time of entry into feedlots most accurately reflects the frequency of *M. haemolytica* colonization, due to increased shedding resulting from stress associated with transportation (Frank and Smith, 1983). Our data suggest that this may not be the case and that a large number of cattle can actively shed *M. haemolytica* after ≥60 days in the feedlot.

Studies on antimicrobial use and development of resistance in bacteria from livestock have focussed mainly on the potential impact on human health (Marshall and Levy, 2011). Resistance in veterinary pathogens has received less attention, despite the implications not only for animal health and welfare but also for the increased use of antimicrobial agents as a result of failed therapy. Given the few antimicrobial agents available to treat BRD (Portis et al., 2012), it is important to monitor the effect of commercial practices on resistance in BRD-related bacteria including *M. haemolytica*.

Metaphylactic treatment of cattle at high-risk of developing BRD upon feedlot entry has shown efficacy in reducing the rates of BRD (Taylor et al., 2010). Effective products include ceftiofur, florfenicol, tulathromycin, and tilmicosin (Taylor et al., 2010). Tulathromycin was approved for use in Canada in 2006 to treat and prevent BRD in feedlot cattle (Schunicht et al., 2007) and was accordingly being used by the feedlots enrolled in the present study. Sampling of cattle was random and took place over a 3-year period shortly after the approval of tulathromycin. Our study therefore represents a baseline of tulathromycin susceptibility in the BRD pathogen* M. haemolytica* in the feedlots analyzed.

More than 99% of *M. haemolytica* were susceptible to a tulathromycin concentration of 2 μg/mL, which is several magnitudes less than the resistant breakpoint (64 μg/mL). Five isolates were capable of growing on BHI plates supplemented with 2 μg/mL tulathromycin and each of these isolates were later confirmed as tulathromycin-resistant, having MICs of 64 μg/mL. We selected a concentration of 2 μg/mL for screening isolates with reduced tulathromycin susceptibility based on one of the few studies that have reported tulathromycin MICs in *M. haemolytica* field isolates in Europe (Godinho, 2008). Regulations in the European Union allow for tulathromycin to be used to prevent BRD, though unlike prevention in Canada, only after confirmation of the disease in a herd has been established (European Medicines Agency, 2013). Similar to our study, Godinho (2008) reported that low levels (97%) of bovine *M. haemolytica* field isolates had tulathromycin MICs of ≤2 μg/mL and a narrow range of MICs.

Of the 1,688 individual cattle positive for *M. haemolytica*, 18.5, 2.9, and 2.4% were administered therapeutic concentrations of tulathromycin, tilmicosin, or tylosin tartrate, respectively. In addition, 13.2% were administered subtherapeutic concentrations of tylosin phosphate in feed. We included in our analyses all macrolides commonly used in feedlot production because some tulathromycin resistance genes have also been shown to confer resistance to tilmicosin and tylosin (Rose et al., 2012) and increase the MICs of tildipirosin and gamithromycin (Michael et al., 2012a). Theoretically, tilmicosin and tylosin could have therefore exerted a selection pressure for tulathromycin resistance. Our study found no evidence for this relationship, as tulathromycin-resistant *M. haemolytica* were only isolated from three cattle. While it is interesting to note that all three of these animals received metaphylactic doses of tulathromycin and that resistant *M. haemolytica* were isolated only at ≥60 days on feed, these remarkably low rates of resistance do not support any association between macrolide use and tulathromycin resistance. It appeared that the antimicrobial administration practices used by commercial feedlots in the present study did not select for tulathromycin resistance over 3 years of sampling. This is supported by a study that measured no detectable changes in resistance in *M. haemolytica* from feedlot cattle 28 days after therapeutic administration of tulathromycin and tilmicosin, or subtherapeutic administration (in-feed) of tylosin (Zaheer et al., 2013).

We focussed only on tulathromycin resistance owing to its rapid adoption by industry and the opportunity to measure changes in resistance after introduction of a new antimicrobial prescribed for metaphylactic and therapeutic use in feedlot cattle. The percent of tulathromycin-resistant *M. haemolytica* increased from 0% in year one to 0.4% in year three of our study. Tulathromycin-resistant *M. haemolytica* were only detected in the third year, and this corresponds to 4 years after the approval of tulathromycin in Canada (Health Canada, 2006). Few other studies have reported on temporal antimicrobial susceptibility in *M. haemolytica* since the approval of tulathromycin for preventing or treating BRD. Over a similar 3-year timeframe, the German national monitoring program GE*RM*-VET reported an increase in tulathromycin resistance in *M. haemolytica* from undetectable in 2004/2005 in 131 isolates examined, to 2% of 55 isolates that were tested in 2006/2007 (BVL, 2011). A comprehensive study analyzed resistance in *M. haemolytica* isolated from diseased or deceased cattle that were processed from 24 veterinary diagnostic labs across the United States and Canada (Portis et al., 2012). More than 300 isolates per year for the years 2004 through 2009 were analyzed. The authors identified tulathromycin-resistant *M. haemolytica* in 2004, the year before the drug was approved for use in the United States and 2 years before approval in Canada. The reported percentages of resistant isolates were 1.8, 2.4, 7.1, 10.7, 9.5, and 8.9 from years 2004–2009, respectively. The large increase in resistance after the introduction of tulathromycin is in contrast to our study. This difference likely reflects the population of animals used in the two studies. The study by Portis et al. (2012), included sick and diseased animals whereas our study was a random sample of primarily healthy cattle in feedlots. There is strong evidence to suggest that *M. haemolytica* serotypes 1 and 6 are most frequently associated with BRD (Rice et al., 2008), and also carry the majority of antimicrobial resistance determinants within the *M. haemolytica* population (Katsuda et al., 2013; Klima et al., 2013). Reasons for this are unclear but may be related to increased antimicrobial use in morbid animals infected with serotypes 1 and 6, and thus increased resistance in these particular serotypes. In a recent study however, no *M. haemolytica* (*N* = 41) isolated from cattle diagnosed with BRD in southern Alberta from 2007 to 2008 were resistant to tulathromycin (Klima et al., 2013). The differences in these studies are difficult to explain but may also be affected by geography or feedlot management.

Each of the tulathromycin-resistant *M. haemolytica* isolates were serotype 1 and multi-drug resistant. This supports previous observations that multidrug resistance is more likely to occur in serotype 1 isolates in Canadian feedlots (Klima et al., 2011, 2013). Only two pulsotypes were observed in the bacteria, with all strains being closely related and having a maximum of three bands difference in restriction patterns (Tenover et al., 1995; **Figure 1**). Three of the isolates (32A, 32B, 32C) originated from a single animal and appeared to be clones. Isolates 50A and 55A were collected from separate cattle, but also appeared to be clonal, having identical pulsotypes. The animals carrying isolates 50A and 55A were housed in the same pen and it is possible that transmission of the strain occurred between these two individuals while in the feedlot. Horizontal transfer of *M. haemolytica* between cattle in beef operations has previously been reported (Briggs et al., 1998; Timsit et al., 2013). In addition to tulathromycin, each isolate was resistant to neomycin, oxytetracycline, and tilmicosin and all possessed genes conferring resistance to neomycin (*aphA-1*) and oxytetracycline (*tet*(H)). Oxytetracycline and neomycin resistances are the most common types of resistance in *M. haemolytica* in Canadian feedlots (Klima et al., 2011). Isolates 55A and 50A also encoded *bla*_ROB-1_, conferring resistance to penicillin and ampicillin however only 55A was resistant to these β-lactams. This implies that isolate 50A somehow lost functionality for ampicillin resistance, despite having a resistant genotype. None of the isolates carried any of the macrolide resistance genes that were tested.

The genes *erm*(42) and *msr*(E)-*mph*(E) confer macrolide-lincosamide and macrolide-triamilide resistance, respectively (Kadlec et al., 2011). They have been identified as part of the integrative conjugative element ICEPmu1 in *Pasteurella multocida* (Michael et al., 2012b) and have been shown to conjugatively transfer from *P. multocida* to *M. haemolytica* (Michael et al., 2012c). Recently, *erm*(42) and *msr*(E)-*mph*(E) have been identified in *M. haemolytica* isolates collected from cattle in the United States and appear to have been acquired from other members of *Pasteurellaceae* (Desmolaize et al., 2011). We have also observed the same genes on mobile genetic elements in *M. haemolytica* isolated from deceased cattle in the United States (Klima et al., 2013). Their absence in any of the isolates of the current study could again be due to geographical differences or the population of cattle investigated. No other genes have yet been reported to confer macrolide resistance in *M. haemolytica*. The isolates in our study may encode a novel tulathromycin resistance gene. We are currently in the process of sequencing the genomes of these isolates to explore this possibility. With the isolates being closely related, it is likely that the resistance element was only recently acquired. Clonal expansion of fluoroquinolone-resistant *M. haemolytica* amongst cattle with BRD has been observed (Katsuda et al., 2009). Regardless, expansion of tulathromycin-resistant *M. haemolytica* in the feedlots we analyzed seemed limited, due to the infrequent observation of this resistant phenotype.

In conclusion, the current study showed that tulathromycin resistance in *M. haemolytica* from a general population of feedlot cattle in western Canada was exceptionally low even after this antibiotic had been used in the industry for a period of 4 years. There was no evidence that the commercial practices used by the feedlots in this study selected for tulathromycin-resistant *M. haemolytica*. However, resistance has been observed to increase in isolates from morbid or deceased cattle. These differences may be due to geography, commercial practices, or the repeated antimicrobial treatment of morbid cattle. Future research should include healthy cattle and those diagnosed with BRD from the same feedlots to better understand selection of resistant *M. haemolytica*.

## Conflict of Interest Statement

The authors declare that the research was conducted in the absence of any commercial or financial relationships that could be construed as a potential conflict of interest.
